# MyD88 inhibitor TJ-M2010-5 alleviates spleen impairment and inflammation by inhibiting the PI3K/miR-136-5p/AKT3 pathway in the early infection of *Trichinella spiralis*

**DOI:** 10.1186/s13567-025-01459-2

**Published:** 2025-02-04

**Authors:** Huifang Bai, Qianqian Dang, Guoliang Chen, Lingfeng Xie, Saining Wang, Ning Jiang, Xiaoxia Wu, Shuyan Zhang, Xuelin Wang

**Affiliations:** State Key Laboratory for Diagnosis and Treatment of Severe Zoonotic Infectious Diseases, Key Laboratory for Zoonosis Research of the Ministry of Education, Institute of Zoonosis, and College of Veterinary Medicine, Jilin University, Changchun, 130062 China

**Keywords:** TJ-M2010-5, MyD88, inflammation, *Trichinella spiralis*, methylation

## Abstract

*Trichinella spiralis* (*T. spiralis*) has been reported to induce inflammation, which can cause immune system dysregulation. Myeloid differentiation primary response gene 88 (MyD88) is implicated in inflammation signalling pathways. TJ-M2010-5 is a novel MyD88 inhibitor with remarkable protective effects against several diseases. However, the precise mechanism of TJ-M2010-5’s involvement in spleen impairment and inflammation in the early infection of *T. spiralis* has yet to be fully elucidated. This study analysed histological, inflammation, and macrophage polarisation of the early *T. spiralis*-infected mice treated with TJ-M2010-5. MyD88 promoter methylation results showed that the methylation levels in the 5 d group were lower compared to the control group (*P* < 0.05). Furthermore, the methylation led to an imbalance in anti-inflammatory regulation in the infected mice. After TJ-M2010-5 treatment, spleen impairment was reduced. Sequencing analysis showed that TJ-M2010-5 significantly up-regulated 9 and down-regulated 10 miRNAs compared with the 5 d group. A dual-luciferase reporter assay further revealed that miR-136-5p is involved in the TJ-M2010-5 treatment by targeting AKT3. In RAW264.7 cells, TJ-M2010-5 pre-treatment significantly reversed the M1 polarisation and inhibited nitric oxide (NO) production. LC–MS/MS results showed TJ-M2010-5 was hepatosplenic-targeted. In conclusion, the study demonstrates that TJ-M2010-5 could effectively alleviate spleen impairment and reduce inflammation in mice infected with *T. spiralis* in its early stages by blocking the activation of PI3K/miR-136-5p/AKT3.

**Supplementary Information:**

The online version contains supplementary material available at 10.1186/s13567-025-01459-2.

## Introduction

*Trichinella spiralis* (*T. spiralis*) are foodborne zoonosis parasites and a pathogen of trichinellosis. When infected with *T. spiralis*, the host experiences chronic inflammation for a prolonged period and exhibits immune disorders [1]. The host can also experience two types of tissue damage: direct damage caused by the pathogen and immunopathological damage. The spleen is a vital peripheral immune organ comprising an abundance of immune cells, including T and B lymphocytes, monocytes, macrophages, and dendritic cells. It plays a crucial role in the onset and progression of various diseases. However, spleen impairment and its effect on immune regulation remain unknown during the early infection of *T. spiralis*.

Studies have shown that initiating immune responses in resisting pathogens is closely correlated with toll-like receptors (TLRs) [2]. As pro- or anti-inflammatory molecules and signalling molecules in the TLR pathways, they can activate the host’s innate immune system to recognise various pathogens and defend against their invasion. Toll/IL-1R (TIR) is an essential cytoplasmic adaptor molecule coupled with myeloid differentiation primary response gene 88 (MyD88) [3]. MyD88 can interact with TIR domains to recognise various TLRs without TLR3.

In one study, alleviating inflammation was achieved by delaying the assembly of MyD88 and TLR4 during *T. spiralis* infection [4]. Ma et al. reported that *T. spiralis* galectin (Tsgal) binding to TLR4 in the gut epithelium activated the MAPK-NF-κB pathway, induced the expression of TLR4 and pro-inflammatory cytokines, induced intestinal inflammation, and mediated larval invasion of the gut mucosa [5]. Furthermore, according to another study, galactomannan can bind to recombinant *T. spiralis* galectin (rTsgal), inhibiting the ability of rTsgal to facilitate larval invasion of intestinal epithelial cells (IECs) in vitro. This process was influenced by the polarisation of macrophages towards an M1 phenotype, resulting in increased antibody-dependent cytotoxicity (ADCC) against newborn larvae (NBL) [6]. Macrophages are not only rich in TLR4 receptors on their surface but also play an important role in the immune response to parasitic infections [7].

It has also been shown that rTsCatL2 (recombinant *T. spiralis* cathepsin L domains)-induced M1-type RAW264.7 results in the death of *T. spiralis* NBL [8]. Furthermore, rTsDPP1 (recombinant *T. spiralis* dipeptidyl peptidase 1) promotes macrophage polarisation towards the M2 phenotype. This promotion increases the expression of anti-inflammatory cytokines and suppresses macrophage-mediated ADCC by activating the STAT6/PPARγ pathway. This mechanism facilitates the parasitism and immune evasion of the nematode [9]. Therefore, it is essential to study the role of macrophages in the early infection of *T. spiralis*.

Further studies have suggested that DNA methylation and noncoding small RNAs (18 ~ 22 nucleotides) can contribute to a coordinated epigenetic control of gene expression [10]. Regulating gene expression at transcriptional and posttranscriptional levels can involve pathogen infection [11, 12]. Furthermore, microRNA (miRNA) is a vital regulator in down-regulating drug function genes [13]. Of particular interest, one study demonstrated that noncoding small RNAs, including miRNAs, can regulate the development of parasitic diseases [14]. However, the miRNA regulation mechanism was still not shown during the early infection of *T. spiralis*. As a novel MyD88 inhibitor, TJ-M2010-5 has remarkable protective effects in treating colitis, liver fibrosis, renal interstitial fibrosis, and myocardial ischaemia/reperfusion injury [15, 16]. However, the precise molecular regulatory mechanisms of TJ-M2010-5 and its regulation by miRNAs in the development of inflammatory diseases caused by early *T.* spiralis infection are poorly understood*.*

This study aimed to evaluate the protective role of TJ-M2010-5 on spleen impairment and inflammation in early *T. spiralis*-infected mice. To assess spleen impairment and inflammation, we examined histological changes and macrophage polarisation. Furthermore, we evaluated the methylation rate of the MyD88 promoter in spleen tissues from mice infected early with *T*. *spiralis*. Microarray analysis was used to explore the expression profiles of miRNAs in spleen tissue, focusing on miR-136-5p, which was considered a promising candidate for regulating inflammation in the TJ-M2010-5-treated group. In conclusion, we intended to reveal that TJ-M2010-5 up-regulated miR-136-5p levels, changing the imbalance in anti-inflammatory regulation induced by the MyD88 promoter methylation and alleviating inflammatory responses by involving the PI3K/AKT3 pathway.

## Materials and methods

### Animals, parasites, drugs and tissue collection

The female BALB/c mice (weight 18 ± 2 g) were purchased from the Experimental Animal Center of College of Basic Medical Sciences, Jilin University (Changchun, China). All mice were raised and handled according to the National Institutes of Health guidelines for the care and use of laboratory animals and were approved by the Ethics Committee for Animal Experiments of Jilin University (NO. 202201-104, date: 25/2/2022).

*T. spiralis* (ISS534 strain) muscle larvae (ML) were maintained by serial passage in ICR mice. ML was used to orally infect mice with 250 larvae each for the infection groups. TJ-M2010-5 (MedChemExpress, Princeton, NJ, USA) was dissolved in dimethyl sulfoxide (DMSO) at 50 ℃ with continuous stirring for 30 min. For the protein expression of MyD88 in spleen tissues, the animals were divided as follows (three mice per group): 0 d group: non-infected mice; 3 d, 5 d, 9 d, and 20 d group: infected mice sacrificed at 3, 5, 9, 20 days post-infection (dpi), respectively.

For other animal experiments, the mice were divided into three groups (three mice per group): Group i (0 d): non-infected mice; Group ii (5 d): infected mice sacrificed at 5 dpi; Group iii (TJ5_5 d): infected mice, the first administration on the second day after infection, followed by an intraperitoneal injection TJ-M2010-5 for five successive days (30 mg/kg/day) and sacrificed at 5 dpi. Peripheral blood samples from the mice were collected to separate the sera and were stored at −80 ℃. Spleen samples were also obtained for further experiments.

For tissue distribution experiments, fifteen mice were randomly divided into five groups (three mice per group). The mice in each group were sacrificed at 0.5, 2, 6, 12, and 48 h after receiving an intraperitoneal injection of TJ-M2010-5 (30 mg/kg). Subsequently, major organs were acquired: liver, spleen, lung, and kidney. Tissues were weighed, homogenised in 70% acetonitrile and stored at −80 ℃ to analyse.

### Cell culture

The mouse macrophage cell line RAW264.7 and the human embryonic kidney 293 T cell line HEK 293 T were purchased from the Culture Collection of the Chinese Academy of Sciences (Shanghai, China). RAW264.7 cells were cultured in RPMI 1640 medium (Gibco, Grand Island, NY, USA) at 37 ℃ in a humidified incubator containing 5% CO_2._ HEK 293 T cells were grown in DMEM/High-Glucose culture medium (Gibco, Grand Island, NY, USA) per the above process.

### Histological analysis

The spleen tissue samples collected from the grouped mice were fixed with 4% paraformaldehyde. Following dehydration, these tissues were embedded in paraffin and cut into 5 μm slices to stain with haematoxylin and eosin (H&E) (Solarbio, Beijing, China). Images of the stained cells were visualised by the microscope (OLYMPUS TH4-200, Olympus, Tokyo, Japan).

### Bisulfite sequencing PCR (BSP)

Genomic DNA was extracted using the EZ DNA Methylation Gold™ kit (Zymo Research, Orange, CA, USA). The NanoDrop 2000 Spectrometer (Thermo Scientific, Waltham, MA, USA) was used to detect DNA quantity and purity (A260/A280 ratio). The study intercepted the transcription start site of the 2000-bp gene upstream of MyD88 in the promoter region of the MyD88 CpG islands (CGIs). PCR was performed using HotStar Taq polymerase (Takara, Beijing, China). PCR used the primers (Forward: 5′- GAGGGGTTTTATTTTGAAGTTTTTA -3′, Reverse: 5′- CCTATTCCTAAAACTCTACACCCAA -3′). The primers used were designed using the online MethPrimer software. The PCR products were electrophoresed in 1% agarose gels and visualised by ultraviolet illumination.

### DNMTs and TETs activity assay

Cytoplasmic and nuclear proteins from spleen tissues were extracted using a nuclear extraction kit (Solarbio, Beijing, China). According to the instructions, the enzyme activity of DNMTs (DNA methyltransferases) and TETs (ten-eleven translocation proteins) was assessed using DNMTs activity assay colourimetric kit (Epigentek, Farmingdale, NY, USA) and TETs activity assay colourimetric kit (Epigentek). The optical density (OD) values in the assay were obtained by BioTek Cytation5 with Gen5 software (Agilent, Santa Clara, CA, USA).

### Macrophage M/M2 ratio analysis in spleen tissues by flow cytometry

Suspensions of single cells from the spleen tissues of the experimental mice were prepared. The cells were stained using anti-mouse F4/80 PE (BioLegend, San Diego, CA, USA), anti-mouse CD86 FITC (BioLegend) and anti-mouse CD206 APC antibodies (Invitrogen, Waltham, MA, USA) at 37 ℃ for 30 min. Cells were detected and analysed by FACSCalibur (BD Biosciences, San Diego, CA, USA) and FlowJo software (TreeStar, Ashland, OR, USA), respectively.

### Enzyme-linked immunosorbent assay (ELISA)

The concentrations of interleukin (IL)-4, IL-13, IL-12p70, and TNF-α in serum were quantified using an ELISA kit (Proteintech Group, Chicago, IL, USA) according to the manufacturer’s guidelines.

### miRNA expression profiling

The differentially expressed miRNAs (DEmiRNAs) were analysed by miRNA sequencing as previously described [17, 18]. The miRNA library and deep sequencing were then constructed by Novogene Bioinformatics Technology Co., Ltd (Beijing, China) and DESeq 2 (1.14.1) was used to conduct the differential expression analysis [19]. Venn diagrams, volcano plots, and heatmaps of DEmiRNAs were drawn by the R package (version 3.5.1, New Zealand, University of Auckland). Finally, the change in the expression of the selected miRNAs was verified by quantitative real-time PCR (qPCR). The specific primers are shown in Additional file 1.

### miRNA target genes prediction and validation

We used miRDB and TargetScan7.2 to predict the target genes of miR-136-5p. The overlapping predicted genes were selected for further analysis. The AKT3 gene’s wild-type (WT) or mutated-type (MUT) 3' untranslated region (UTR) was successfully inserted into the pmirGLO luciferase reporter vector (Promega, Madison, WI, USA). The recombinant plasmids were purified using the EndoFree Plasmid Maxi Kit (Vazyme, Nanjing, China). The HEK 293 T cells were co-transfected with miR-136-5p mimics or mimics negative control (NC) and AKT3 WT plasmid or AKT3 MUT plasmid using Lipo2000 transfection reagent (Promega, Madison, WI, USA). This transfection process was carried out for 24 h. For this experiment, there were four groups, including miR-136-5p mimics + AKT3 WT, mimics NC + AKT3 WT, miR-136-5p mimics + AKT3 MUT, and mimics NC + AKT3 MUT. The Dual-Glo luciferase reporter assay system (Beyotime Biotechnology, Haimen, China) was used to assay the luciferase activity after the transfection of plasmids.

### Cell viability assay

TJ-M2010-5 (0, 5, 10, 20, 30, 40, 50 μM) were pre-treated with RAW264.7 cells. The viability of the cells was determined using a CCK-8 kit (Solarbio, Beijing, China), following the manufacturer’s instructions. After culturing for 24 h, a CCK-8 kit was used again to assay the cell viability and the OD value at 450 nm was measured.

### Total RNA extraction and quantitative polymerase chain reaction

Total RNA from cells was extracted using TRIzol reagent (Invitrogen, Waltham, MA, USA). RNA concentrations were determined using a NanoDrop™ 2000 (Thermo Fisher Scientific, Waltham, MA, USA). According to the protocols, all reactions were conducted by ABI 7500 real-time PCR system (Applied Biosystems, Thermo Fisher Scientific, Waltham, MA, USA). The 2^−ΔΔCT^ method was used to calculate the comparative threshold cycle (CT) value. The specific primers are shown in Additional file 1.

### Nitric oxide assay

RAW264.7 cells pre-treated with TJ-M2010-5 were incubated for 6 h. The culture medium was supplemented with LPS (1 μg/mL). After incubation for 24 h, NO production was analysed using the Griess Reagent System (Beyotime Biotechnology, Haimen, China).

### Macrophage M/M2 ratio analysis in RAW264.7 cells by flow cytometry

RAW264.7 cells were stained using anti-mouse F4/80 PE (BioLegend), anti-mouse CD86 FITC (BioLegend), and anti-mouse CD206 APC antibodies (Invitrogen, Waltham, MA, USA) at 37 ℃ for 30 min. Cells were detected and analysed by FACSCalibur (BD Biosciences, San Diego, CA, USA) and FlowJo software (TreeStar, Ashland, OR, USA), respectively.

### Western blot

Cell and tissue lysis was performed using a radioimmunoprecipitation assay (RIPA) lysis buffer (Solarbio, Beijing, China). The cytosolic and nuclear extracts were prepared as previously described. Proteins were isolated by sodium dodecyl sulfate–polyacrylamide gel electrophoresis (SDS-PAGE) and transferred onto nitrocellulose filter (NC) membranes (Millipore, Billerica, MA, USA). NC membranes were blocked with 5% skim milk for 1 h at 37 ℃ and sustained shaking. After washing with Tris-buffered saline and Tween 20 (TBST) thrice, NC membranes were incubated overnight with primary antibodies at 4 °C. After incubating with secondary antibodies for 1 h at 37 °C and washing, we detected the immunoreactive bands using the Millipore chemiluminescent HRP reagent. Protein bands were recorded with a UVP ChemStudio (Analytik Jena, Germany). All antibodies are listed in Additional file 2.

### Liquid chromatography–tandem mass spectrometry (LC–MS/MS)

For the linearity curve generation, TJ-M2010-5 in DMSO was diluted with ddH_2_O to make serial concentrations of the calibration standards solutions (50, 100, 200, 400 and 1000 ng/mL). Subsequently, 1 μL of the supernatant from the tissue homogenates (liver, spleen, lung, kidney) was injected into the LC–MS/MS system.

### Statistical analysis

All experiments were repeated thrice. Statistical analysis was executed using the SPSS 20.0 software package (SPSS Inc., Chicago, IL, USA), and data were presented as mean ± SEM. GraphPad Prism 8.0 software (GraphPad Software, San Diego, CA, USA) was used to display the data. The Shapiro–Wilk test was employed to test the normality of the data. The differences between the two groups were evaluated using ANOVA analysis or Student’s *t*-tests. The analysis of the categorical variables was conducted using the Mann–Whitney test. *P* < 0.05 was considered statistically significant.

## Results

### MyD88 up-regulation and spleen impairment in the early *T. spiralis*-infected mice

**Figure 1:**
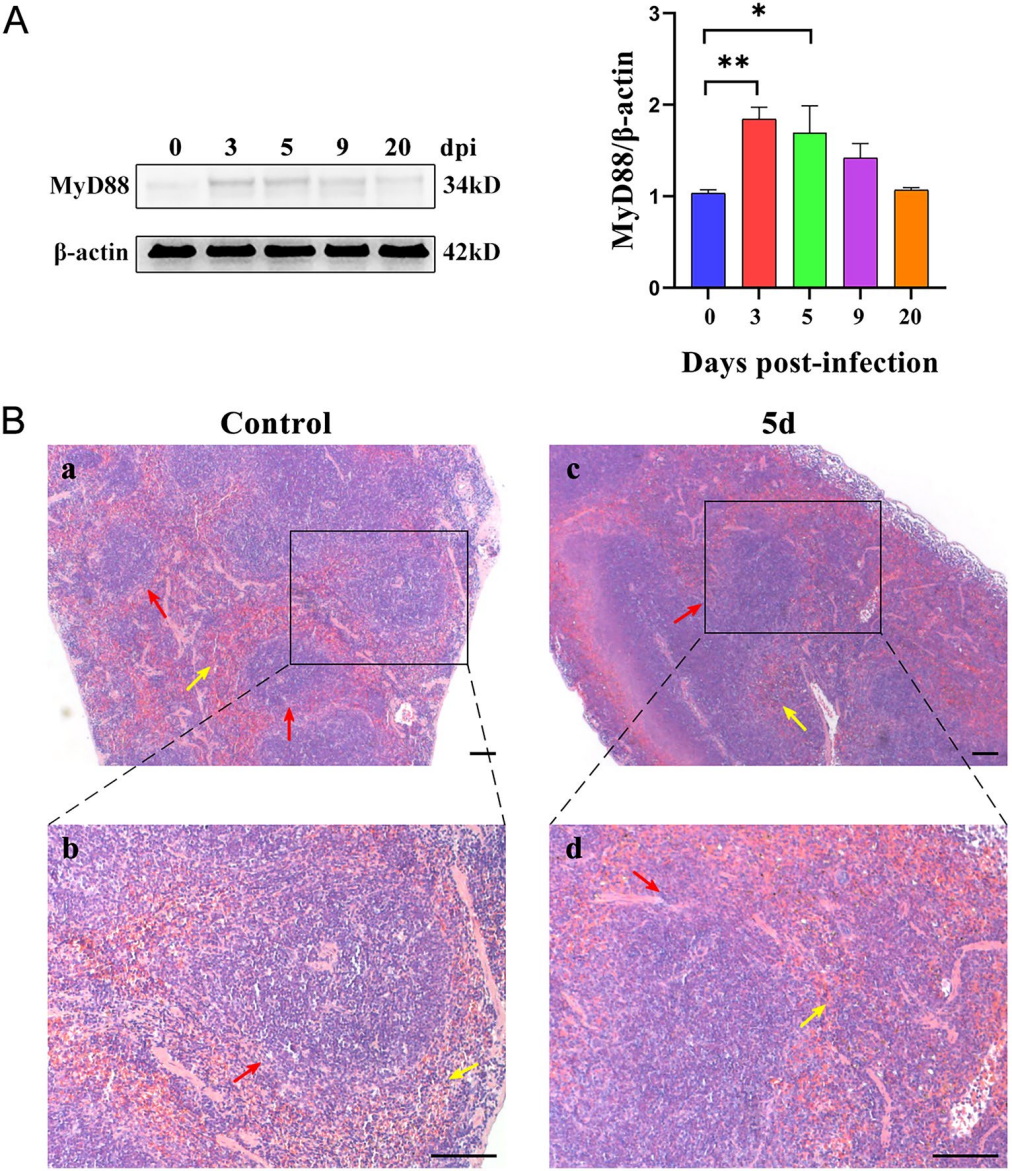
**MyD88 upregulation and spleen impairment in the early *T. spiralis*-infected mice**. **A** MyD88 protein expression in mice spleen was quantified by western blotting on 0, 3, 5, 9, and 20 dpi, respectively. The protein intensity was analysed by using ImageJ. *P < 0.05, **P < 0.01, ***P < 0.001. **B** (**a**) and (**b**) control group. (**c**) and (**d**) 5 d infected group. red arrows: white pulp; yellow arrows: red pulp; Scale bar = 100 μm.

The western blot results for the 3 d and 5 d groups indicated a significant increase in MyD88 protein levels compared to the 0 d group (*P* < 0.05, Figure 1A). The results signified that *T. spiralis* infection could up-regulate the protein expression of MyD88. Therefore, for the study on early *T. spiralis* infection, we used 5 d to represent the infected stage. Histological results showed that the spleen presented normal morphology, with a clear red pulp (RP) and white pulp (WP) for the control group. There was also a dense distribution of lymphocytes in RP and WP (Figure 1B). The structure of the spleen corpuscles was damaged in the 5 d group. The interface between WP and RP was blurred, and its arrangement was sparse. Furthermore, the number of lymphocytes was relatively low. Meanwhile, RP displayed hyperplasia (Figure 1B).

To investigate the protective effect of TJ-M2010-5 against pathological damage caused by early *T. spiralis* infection, we conducted histological analysis on the TJ5_5 d group. We found that pre-treatment with TJ-M2010-5 partly reversed these alterations in the WP and RP (Additional file 3). Our findings demonstrate that the up-regulation of MyD88 is a key contributor to the impairment of the spleen caused by trichinella infection.

### MyD88 promoter methylation modification and DNMTs/TETs activity

**Figure 2:**
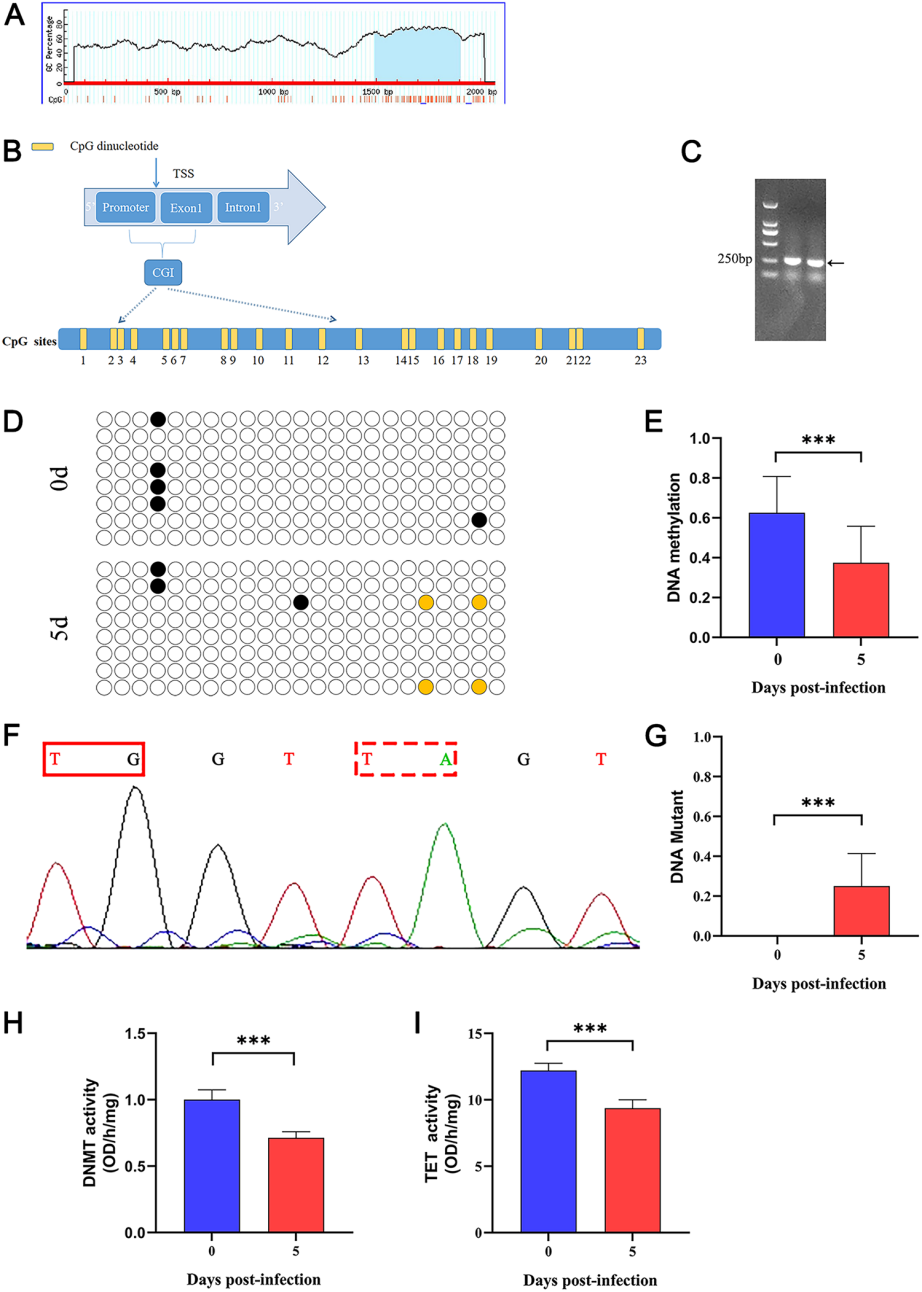
**Methylation levels of the MyD88 promoter in mice challenged with** ***T. spiralis* and DNMTs and TETs activities assays**. **A** MethPrimer software prediction of CGI of the MyD88 promoter region. The blue areas on the picture indicate the potential CGI. **B** The distribution of CpG dinucleotides in the CGI of MyD88. The CGI has a cluster of 23 individual CpG sites, and each yellow rectangle represents a CpG site. **C** The PCR products of MyD88 promoter with methylation primers. **D**: The methylation status of the promoter of MyD88 on 0 and 5 dpi, respectively. Each row corresponded to a single template DNA molecule cloned. Eight colonies from each ligation were randomly picked and sequenced. Each circle represented a CpG site (black circles represented methylated CpG site; white circles were unmethylated CpG site; yellow circles were mutant CpG site). **E** The methylation level of the MyD88 promoter on 0 and 5 dpi, respectively. **F** The mutant pattern of the CpG site with G/A mutation. The mutation sites measured by sequencing are framed with red. **G** The mutant level of the MyD88 promoter on 0 and 5 dpi, respectively. **H**, **I** Bar charts showing DNMTs (**H**) and TETs (**I**) activities of spleen tissues in 0 and 5 dpi, respectively. *P < 0.05, **P < 0.01, ***P < 0.001.

Previous studies have reported that methylation can alter protein expression levels [20, 21]. Therefore, we analysed the changes in methylation levels in the MyD88 promoter region. MethPrimer software predicted that 23 CpG sites were located in the MyD88 promoter region (Figure 2A and B). The agarose gel electrophoresis showed that PCR products were the same size as the target amplified product (250 bp) (Figure 2C). Moreover, the quantitative methylation analysis of the MyD88 promoter revealed that the methylation level of the 5 d group was lower compared to the 0 d group (*P* < 0.05, Figures 2D and E). Interestingly, aside from the C to T mutation, we also discovered a point mutation from G to A (Figure 2F). The levels of DNA mutation significantly increased by 5 dpi, and there was a significant increase in the 5 d group compared to the 0 d group (*P* < 0.05, Figure 2G).

As previously reported, the DNA methylation patterns occurring in CpG motifs were regulated by DNMTs and hydroxymethylases, including the TETs family of dioxygenases [22]. Our results show that the DNMT activity levels significantly decreased in the 5 d group vs the 0 d group (*P* < 0.05) (Figure 2H). Meanwhile, the TET activity levels significantly reduced in the 5 d group vs the 0 d group (*P* < 0.05, Figure 2I). Moreover, the results suggest that DNMTs and TETs can regulate the methylation of the MyD88 promoter, which could lead to an increase in the expression of MyD88 and the activation of downstream inflammatory signalling pathways.

### TJ-M2010-5 alleviated inflammation and altered the macrophage phenotype in the early *T. spiralis*-infected mice

**Figure 3:**
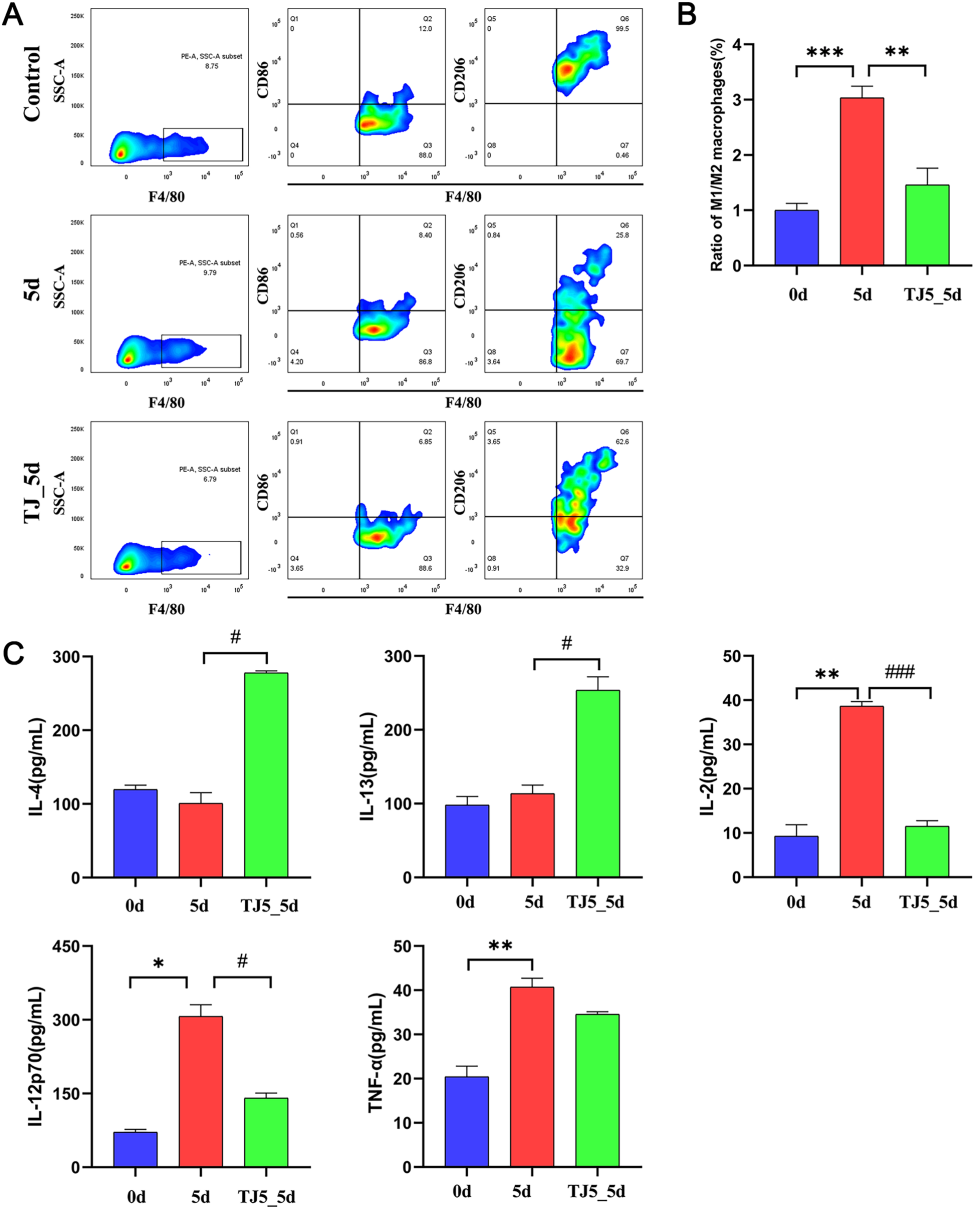
**TJ-M2010-5 protective effect on spleen impairment in the early *T. spiralis*-infected mice.** **A** Representative plots of M1 and M2 macrophages in the spleens of mice. **B** Flow cytometry in different groups determined the M1/M2 ratio. **C** ELISA analysis of IL-4, IL-13, IL-2, IL-12p70 and TNF-α in the serum from different groups. Compared with 0 d group: *P < 0.05, **P < 0.01, ***P < 0.001. Compared with 5 d group: ^#^*P* < 0.05, ^##^*P* < 0.01, ^###^*P* < 0.001.

The methylation status of the current gene cannot be easily altered with existing drugs [23]; therefore, to reduce inflammation and treat diseases, we opted for MyD88, specifically the TJ-M2010-5 inhibitor, to modify the high expression of MyD88. However, it is unclear whether its expression is regulated by adjusting the methylation level. To further reveal the potential mechanism by which TJ-M2010-5 alleviates inflammation in early *T. spiralis*-infected mice, we conducted a polarisation analysis of M1 and M2 macrophages by flow cytometry. Results showed that the ratio of M1 to M2 macrophages in the spleens of trichinella-infected mice was significantly increased (Figure 3A and B). However, TJ-M2010-5 markedly reduced the ratio of M1 to M2. Edictally, Th1 and Th2 cytokines in the serum of the 0, 5, and TJ5_5 d groups were detected, respectively.

Additionally, *T. spiralis* challenge led to a significant increase in the expression levels of IL-2, IL-12p70, and TNF-α and an insignificant change in the expression levels of IL-4 and IL-13 compared with the 0 d group (Figure 3C). However, TJ-M2010-5 treatment reversed the up-regulated levels of Th1 cytokines (except for TNF-α) and down-regulated the levels of Th2 cytokines. These results demonstrate that TJ-M2010-5 substantially alleviated inflammation in the early *T. spiralis*-infected mice and show that the macrophages’ phenotypic switch from M1 to M2 promoted the recovery of the injured spleen.

### TJ-M2010-5 altered miRNA expression profile

**Figure 4:**
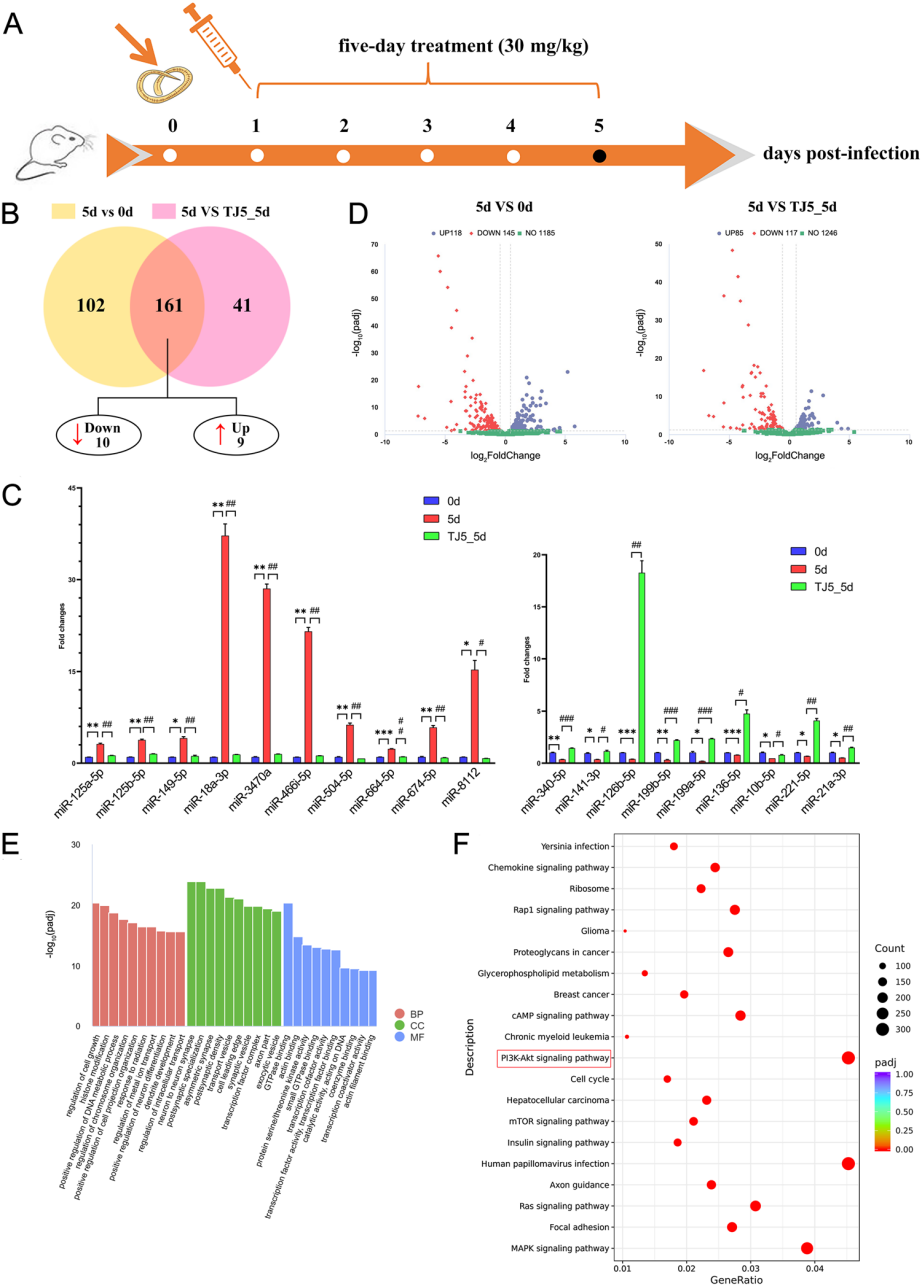
**Global view of miRNAs expression profiles in mice spleens during the early infection of *T. spiralis***. **A**: Schematic diagram illustrating the experimental protocols. B: Venn diagram showing the intersection of the DEmiRNAs among 5 d group vs 0 d group and TJ5_5 d group. C: mRNA expression of DEmiRNAs in different groups (0, 5 and TJ5_5 d groups) by qPCR. Compared with 0 d group: * P < 0.05, ** P < 0.01, *** P < 0.001. Compared with TJ5_5 d group: #* P* < 0.05, ##* P* < 0.01, ###* P* < 0.001. D: Volcano plots of DEmiRNAs in 5 d group vs 0 d group (A) and TJ5_5d group (B). Red, higher-expressed miRNAs; Blue, lower-expressed miRNAs. E–F: GO (E) and KEGG (F) pathway analysis for DEmiRNAs.

As the above results demonstrate, MyD88 was more highly expressed in early trichinella infection (5 d group). Accordingly, this study’s objective was to perform animal experiments to explore the protective role of TJ-M2010-5 in *T. spiralis*-induced inflammation (Figure 4A).

Through miRNA sequencing, we determined that TJ-M2010-5 markedly alters the miRNA expression profile in different groups. Moreover, differential expression analysis showed that TJ-M2010-5 significantly up-regulated 9 and down-regulated 10 miRNAs compared with the 5 d group (Figure 4B). qPCR results indicated that the expression of miR-340-5p, miR-141-3p, miR-126b-5p, miR-199b-5p, miR-199a-5p, miR-136-5p, miR-10b-5p, miR-221-5p, and miR-21a-3p were all significantly up-regulated.

However, miR-125a-5p, miR-125b-5p, miR-149-5p, miR-18a-3p, miR-3470a, miR-466i-5p, miR-504-5p, miR-664-5p, miR-674-3p, and miR-8112 were all significantly down-regulated in TJ5_5 d group vs the 5 d group. These outcomes were consistent with the RNA-Seq dataset (Figure 4C).

A volcano plot (Figure 4D) illustrates all these DEmiRNAs. Gene Ontology (GO) enrichment among the DEmiRNAs revealed that they were mainly enriched in terms of regulating cell growth, neuron-to-neuron synapse, GTPase binding, etc. (Figure 4E). Moreover, KEGG enrichment analysis showed that these miRNAs may be associated with the PI3K/AKT signal pathway (Figure 4F).

### miR-136-5p targeted and regulated AKT3

After analysing the RNA-Seq results and study reports [24, 25], we determined that miR-136-5p may be involved in the TJ-M2010-5 treatment of spleen impairment during the early stages of *T. spiralis* infection.

**Figure 5:**
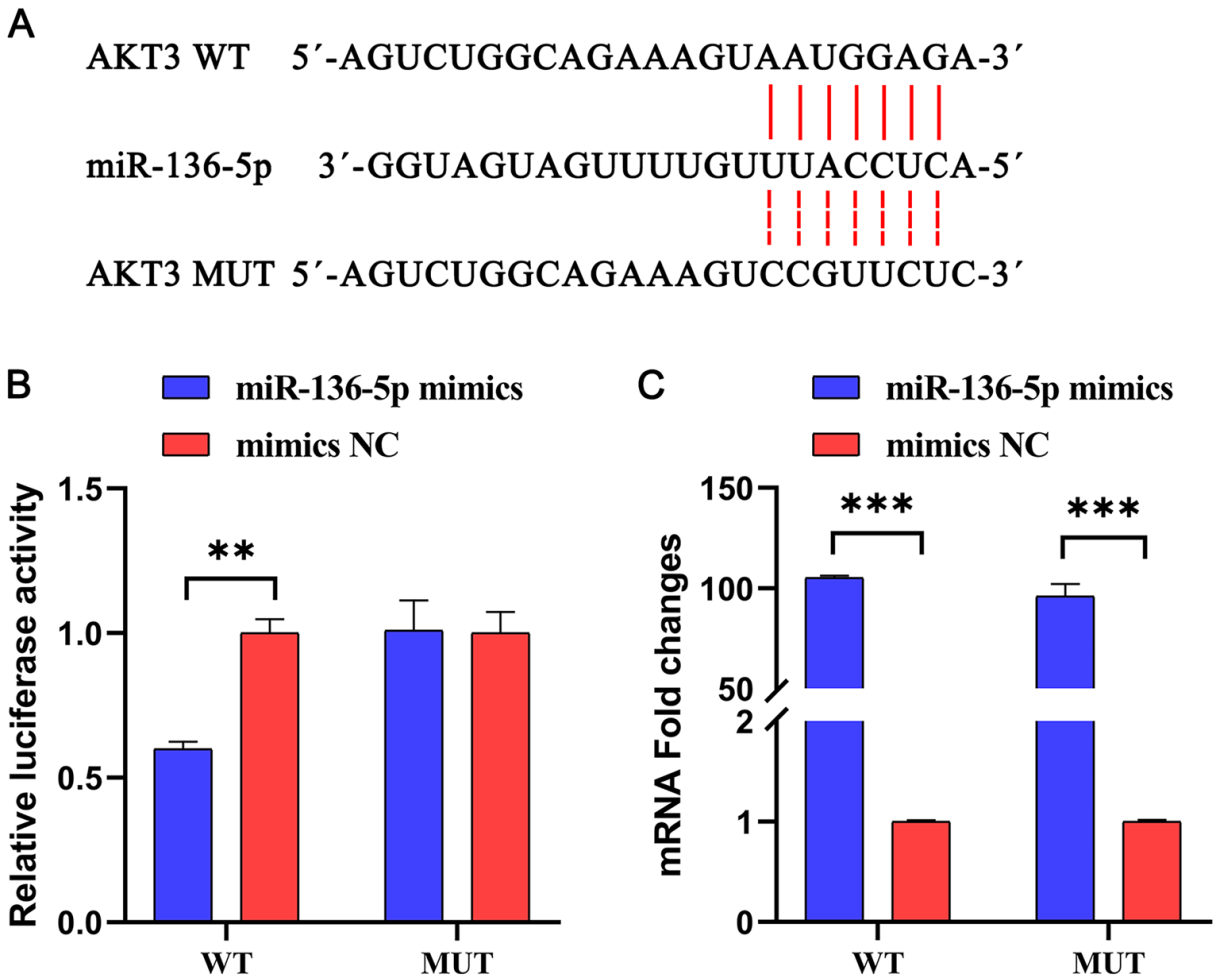
**miR-136-5p targeted and regulated AKT3.** **A** miR-136-5p and complementary AKT3 nucleotide sequence. **B** Assay results of dual-luciferase. **C** Relative expression levels of miR‐136-5p in HEK 293 T cells after transfection with miR‐136-5p mimics and mimics NC.

To identify potential targets of miR-136-5p, we compared its sequence to the mouse genome database. The results showed that the 3′ UTR region of AKT3 contained potential binding sites for the miR-136-5p sequence (Figure 5A). Thus, we transfected miR‐136-5p mimics into HEK 293 T cells to overexpress miR‐136-5p and mimic NC as a control (Figure 5C). The outcomes of the dual-luciferase assay showed that the miR-136-5p mimics + AKT3 WT group suppressed the relative luciferase activity in HEK 293 T cells. However, the miR-136-5p mimics + AKT3 MUT group did not inhibit the relative luciferase activity. These results confirm that AKT3 is the target gene for miR-136-5p (Figure 5B).

### TJ-M2010-5 up-regulated miR-136-5p expression and inhibited the PI3K/AKT3 signalling pathway in RAW264.7 cells

**Figure 6:**
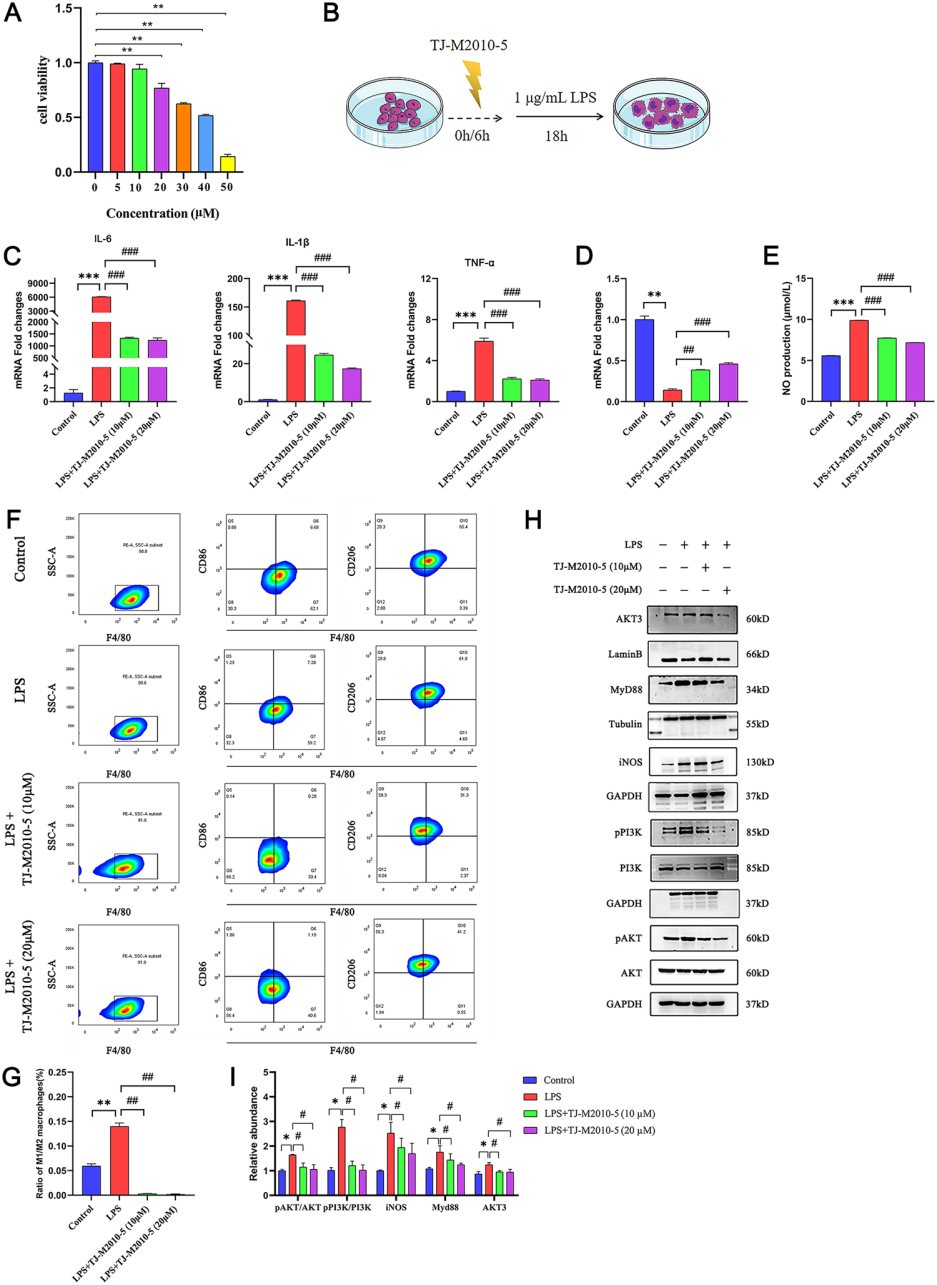
**TJ-M2010-5 reduced inflammation on RAW264.7 cells stimulated with LPS.** **A** CCK8 assay. *P < 0.05, **P < 0.01, ***P < 0.001. **B** Schematic diagram of RAW264.7 inflammatory cell model induced by LPS. **C** IL-6, IL-1β and TNF-α mRNA expression of different groups. **D** miR-136-5p mRNA expression of different groups. **E** TJ-M2010-5 effects on the NO production in RAW264.7 cells stimulated by LPS. **F**, **G** Representative plots of M1 and M2 macrophages pre-treatment with 10 and 20 μM TJ-M2010-5. The M1/M2 ratio was determined by flow cytometry. **H–I** Western blotting analysis of iNOS, pPI3K, PI3K, pAKT, AKT, MyD88 and AKT3 protein expression. The protein intensity was analysed by using ImageJ. Compared with control group: *P < 0.05, **P < 0.01, ***P < 0.001; Compared with LPS group: ^#^*P* < 0.05, ^##^*P* < 0.01, ^###^*P* < 0.001.

The RAW264.7 cells were pre-treated with six concentrations of TJ-M2010-5 (5, 10, 20, 30, 40, 50 μM) for 6 h, and the effect of TJ-M2010-5 on inflammation induced by LPS was analysed (Figure 6B). CCK8 assay showed that 20 μM TJ-M2010-5 had no significant cytotoxicity on RAW264.7 cells (Figure 6A). Therefore, we chose 10 and 20 μM as the concentration in the subsequent experiments.

The results showed that RAW264.7 cells pre-treated with TJ-M2010-5 significantly inhibited LPS-induced inflammation by down-regulating the expression of IL6, IL1β, and TNFα (Figure 6C). LPS down-regulated the expression of miR-136-5p, while TJ-M2010-5 reversed these effects (Figure 6D). Furthermore, the ratio of M1 to M2 macrophages was higher in treatment with the LPS group vs the control group (Figures 6F and G). TJ-M2010-5 pre-treatment showed that it could reverse the M1 polarisation of macrophages, decrease the M1/M2 ratio, and inhibit NO production (Figure 6E).

Western blot revealed that LPS treatment did not affect the total expression of PI3K and AKT but did increase the expression of pPI3K, pAKT, AKT3, MyD88, and iNOS. However, TJ-M2010-5 pre-treatment attenuated this effect (Figures 6H and I). Therefore, TJ-M2010-5 can defend against inflammation and exhibits a prominently protective function by down-regulating the expression of IL6, IL1β, and TNFα. This down-regulating thus reverses the M1 polarisation of macrophages and regulates the PI3K/AKT3 pathways in RAW264.7 cells.

### Tissue distribution

For this study, we used liquid chromatography-tandem mass spectrometry (LC–MS/MS) analysis to quantify the deposition of TJ-M2010-5 in major organs of the mice. The calibration curves, correlation coefficients, and linear ranges of TJ-M2010-5 are provided in Additional files 4–6.

**Figure 7:**
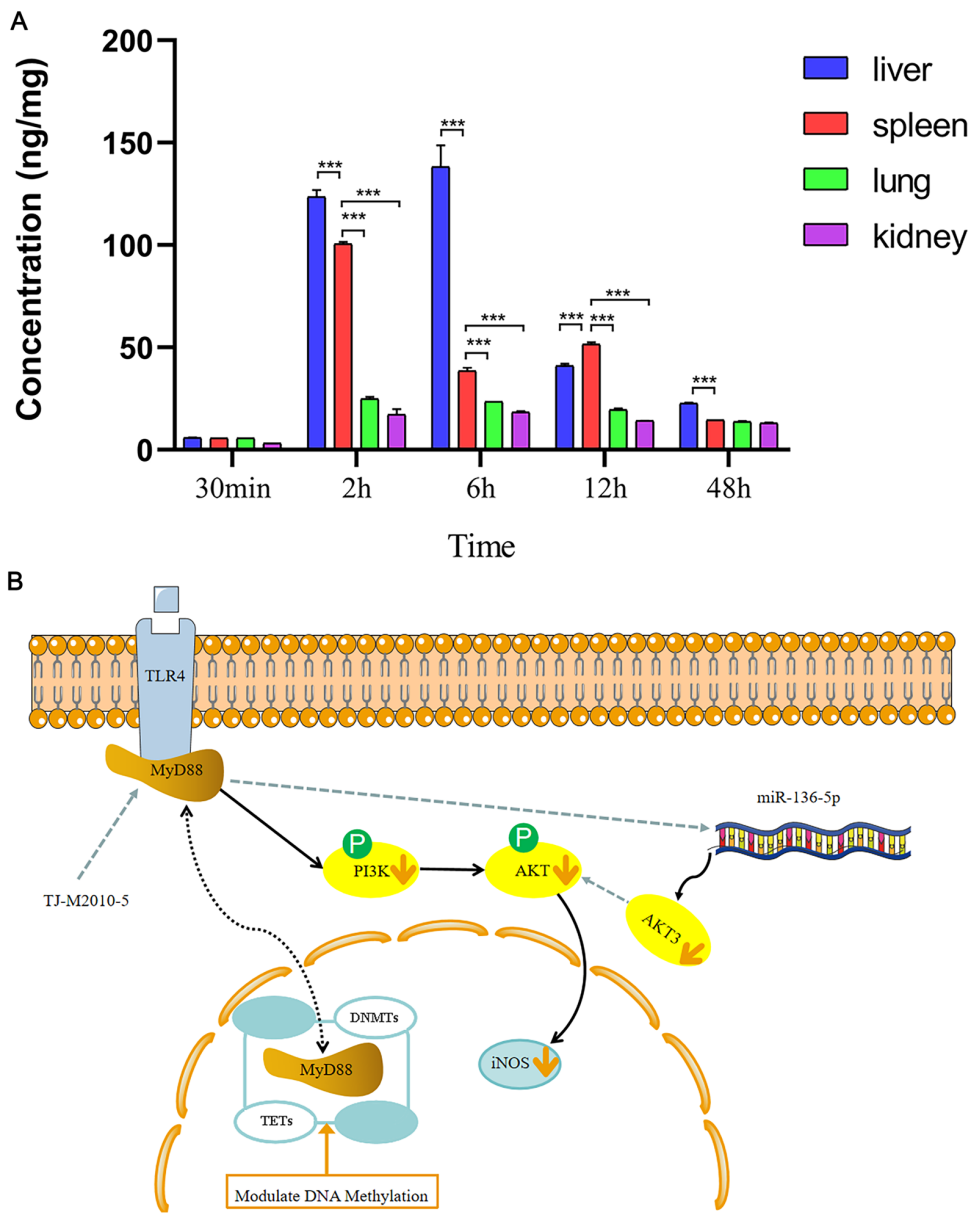
**Tissue distribution and scheme summarising the protective effects of TJ-M2010-5 in spleen impairment**. **A** Concentration of TJ-M2010-5 in different tissues collected at 30 min, 2, 6, 12 and 48 h after 30 mg/kg TJ-M2010-5 treatment. *P < 0.05, **P < 0.01, ***P < 0.001. **B** A schematic diagram of TJ-M2010-5 modulating the inflammatory response via PI3K/AKT3 pathway in spleen impairment.

TJ-M2010-5 presented a good linearity (R^2^ > 0.99), with concentrations between 50 and 1000 ng/mL. At 30 min after administration, the concentration of TJ-M2010-5 in the tissues was ranked high to low as follows: liver > spleen > lung > kidney (Figure 7A).

At 2 h after administration, the concentration of TJ-M2010-5 in the tissues remained in the same order: liver > spleen > lung > kidney. At 6 h after administration, the same concentration order was retained. However, 12 h after administration, the concentration changed to spleen > liver > lung > kidney. At 48 h after administration, the concentration again displayed the order: liver > spleen > lung > kidney. The results suggested that TJ-M2010-5 is hepatosplenic-targeted.

## Discussion

Previous studies have shown that TLR receptors activate the innate immune system and trigger downstream signalling molecules to participate in immune regulation [26]. Han et al. revealed that the MyD88 expression changes during *T. spiralis* infection can affect the secretion of the macrophages inflammatory cytokines and lead to immune suppression [27]. A previous study reported that MyD88-deficient mice display a complete loss in resistance to acute infection of *T. gondii* [28]. Studies have also shown that this adaptor protein’s dysfunction may negatively affect the systemic and local immune response depending on MyD88 expression [29].

Typically, *T. spiralis* infection can activate Th1/Th2 mediated immune response and sustain a long-term inflammatory state in the infection host [30, 31]. In this study, we observed that the spleen tissue of the early *T. spiralis*-infected mice was damaged in the 5 d group. Similarly, we found that the protein expression level of MyD88 was highly expressed in the 5 d group compared with the control group. To further explore this immune imbalance, we assayed the MyD88 promoter region methylation status of spleen tissues after *T. spiralis* infection. Generally, cancer cells have both regional hypermethylation (aberrant DNA methylation) and global hypomethylation [32].

Our study found that MyD88 methylation in mice infected with *T. spiralis* aligned with humans exposed to *H. pylori*–triggered chronic inflammation and colorectal cancer [33, 34]. Previous studies have revealed that regulating gene transcription through DNA methylation is related to the development of various diseases [35–37]. We observed that the methylation status of the MyD88 promoter was significantly decreased in *T. spiralis*-infected mice compared with the 0 d group. Theoretically, the hypermethylation status of DNA sequences is related to the inhibition of gene expression [38, 39].

Interestingly, we also found G to A point mutants. Previous studies have reported that DNA hypomethylation can lead to elevated mutation rates [40, 41], mainly G/A mutations, due to the strong spontaneous deamination and cellular DNA repair machinery [42]. Due to the dynamic transition, gene expression regulation is very sophisticated [43, 44]. Finally, differences in methylation can partially account for the up-regulation of MyD88 protein expression. The changes in the spleen tissues of the early *T. spiralis*-infected mice should be considered.

Furthermore, DNMTs and TETs regulate DNA methylation at specific sites [45, 46]. Importantly, growing evidence indicates that TETs and DNMTs can also alter the expression of important host genes involved in immune responses [47, 48]. In this study, we observed that the enzyme activity of DNMTs and TETs was decreased in the 5 d group vs the 0 d group (*P* < 0.05). Therefore, we hypothesised that the variation in methylation ratios of MyD88 could be partially due to the reduction of DNMTs and TETs function (enzymatic and nonenzymatic activities) [49]. This finding aligns with the findings reported by Zhang et al. [50].

Typically, high MyD88 expression activates the inflammatory signalling pathways after 5 days of trichinella infection [23]. To reduce inflammation and treat the diseases, we selected the MyD88 inhibitor TJ-M2010-5 to interrupt the activation of the inflammatory signalling pathways induced by high MyD88 expression. In the early infection stage, macrophages were predominantly M1, contributing to the inflammatory response and promoting the elimination of worms [8]. Earlier studies have confirmed macrophage TLRs to be involved in pathogen recognition and immune regulation [51]. In our study, a large number of M1-phenotype splenic macrophages were activated after *T. spiralis* infection. TJ-M2010-5 treatment significantly decreased this activation while inducing the transformation of the M1 to the M2 macrophages. TJ-M2010-5 also positively contributed to the decrease of IL-2 and IL-12p70, the up-regulation of IL-4 and IL-13, and the attenuation of the inflammatory response in vivo. However, TJ-M2010-5 did not affect TNF-α levels and insignificantly contributed to the systemic anti-inflammatory response.

Subsequently, we found that TJ-M2010-5 markedly altered the miRNA expression profile in the early *T. spiralis*-infected mice. Notably, the TJ-M2010-5-related differentially expressed miRNAs targeted the PI3K/AKT signalling pathway. Numerous reports have shown that the PI3K/AKT pathway is involved in the mechanisms of inflammation and is a potential target for anti-inflammation therapy [52–54].

Moreover, AKT3 is vital in growth, proliferation, and metabolism and is dysregulated in some oncological diseases [55]. Previous studies have demonstrated that TLR4 initiates the microglia by triggering MyD88 and activating the downstream PI3K/AKT signalling pathway [56]. Yin et al. reported that LPS can remodel the tumour microenvironment and promote the anti-tumour effect via TLR4/MyD88/AKT/NF-κB pathway in pancreatic cancer [57].

However, the role of AKT3 in MyD88-mediated function during early *T. spiralis* infection, specifically in spleen impairment, is poorly understood. Furthermore, miR-136-5p, as a tumour suppressor, plays an important role in regulating various cellular processes [58], inhibiting viability, proliferation, migration, and invasion, and promoting apoptosis of tumour cells [59]. In this study, we detected that the mRNA expression of miR-136-5p was significantly up-regulated in TJ-M2010-5-pre-treated RAW264.7 cells and the spleens of TJ5_5 d mice. The study’s luciferase reporter assays identified AKT3 as a target on miR‐136‐5p. Some drugs or compounds enhanced their anti-inflammatory effects by inhibiting AKT3 expression [60–63].

Macrophages are involved in many parasitic infections [7, 64]. TLR4 on the surface of macrophages plays an important role in innate immunity between parasites and hosts [65–67]. As a TLR4 agonist associated with parasite immunology, the LPS-stimulated macrophage model is ideal and instructive for studying the polarisation of M1 to M2 in vitro.

When LPS is stimulated, macrophages can release a variety of inflammatory factors, such as TNF-α, IL-6, NO, and IL-1β. NO is an important mediator of inflammation amplification. It is produced by nitric oxide synthase (NOS), and the inducible NOS (iNOS) is expressed in various cells, such as macrophages and dendritic cells. NO has assorted biological functions in mammalian cells, including microbial immunity, vasodilation, and the regulation of cell signal transduction [68]. Overproduction of NO may lead to tissue damage and worsen inflammatory conditions. Thus, controlling NO production can alleviate inflammation symptoms. Our investigation indicated that TJ-M2010-5 alleviated inflammation. In addition, it reversed the effect in RAW264.7 cells by activating the PI3K/AKT3 pathway induced by LPS and overexpressing IL-6, IL-1β, TNF-α, NO, and iNOS.

Furthermore, M1 and M2 macrophages are commonly considered pro-inflammatory or anti-inflammatory regulatory properties, respectively. This study showed that TJ-M2010-5 exerted its anti-inflammatory effects by regulating the levels of inflammatory factors and promoting the polarisation of M1 to M2. Consistent with our findings, TJ-M2010-5 can regulate RAW264.7 cell inflammation by inhibiting AKT3 expression. However, from a preliminary perspective of miRNA profile analysis, TJ-M2010-5 affected the expression of multiple miRNAs. Thus, it needs to be investigated whether the effect is produced via additional mechanisms and targets. It is worth mentioning that AKT3 may not be the unique target of miR-136-5p, and more experiments are needed.

In addition, a significant level of TJ-M2010-5 was found in the liver and spleen, likely due to its uptake by the reticuloendothelial system (RES) phagocytic cells [69]. The study showed that TJ-M2010-5 played a negative feedback regulatory role (Figure 7B) in the inflammation signalling pathway. Our outcomes suggest that administering TJ-M2010-5 could facilitate the resolution of the pro-inflammatory state of macrophages and promote the functional improvement of spleen immunity function.

Nonetheless, our research has limitations since DNA methylation differs across cell types within the same tissue. Consequently, MyD88 hypomethylation seems specific to both cell type and tissue in our study. Furthermore, we confirmed that TJ-M2010-5 could reduce inflammation, but we did not verify its involvement in the anti-inflammatory process through the PI3K/AKT3 signalling pathway in vivo experiments. Finally, increasing the sample size of experimental animals is recommended for further research. Therefore, additional, comprehensive studies should be conducted to address these limitations more effectively.

In conclusion, we revealed that TJ-M2010-5 significantly alleviated inflammation in RAW264.7 cells by blocking PI3K/miR-136-5p/AKT3 activation and promoting macrophage polarisation towards M2. The results suggested that MyD88 up-regulation was altered by its methylation in the early *T. spiralis*-infected mice, with DNMTs and TETs being the major enzymes involved. Furthermore, this study demonstrated the protective effect of TJ-M2010-5 in the early *T. spiralis*-infected mice model, which relieved Th1 cytokine secretion, increased Th2 cytokine secretion and promoted splenic macrophage polarisation towards M2. Therefore, we propose that TJ-M2010-5 is a promising therapeutic agent that might provide a clinical alternative for treating inflammation-related diseases caused by parasite infection.

## Supplementary Information

**Additional file 1.** **Primer sequence of target and reference genes.**

**Additional file 2.** **Antibodies used in this study.**

**Additional file 3.** **TJ-M2010-5 protective effect on spleen impairment in T. spiralis infected mice.**

**Additional file 4. Positive mode electrospray ionisation (ESI) mass spectrum of TJ-M2010-5.**

**Additional file 5.** **Total ion chromatogram (TIC) of TJ-M2010-5.**

**Additional file 6.** **Calibration curve, correlation coefficients and linear ranges of TJ-M2010-5.**

## Data Availability

All data generated during this study are included in this published article and its supplementary information files.
