# Supplementary material for: MyD88 inhibitor TJ-M2010-5 alleviates spleen impairment and inflammation by inhibiting the PI3K/miR-136-5p/AKT3 pathway in the early infection of Trichinella spiralis
Source: Vet Res. 2025 Feb 4;56:28. doi: 10.1186/s13567-025-01459-2 (PMC11796171; doi:10.1186/s13567-025-01459-2)
Supplement: Supplementary file 1 — Additional file 1. Primer sequence of target and reference genes. [file 13567_2025_1459_MOESM1_ESM.docx]

**Additional file 1 Primer sequence of target and reference genes.**

| **Primers** | **Sequence (5’ to 3’)** |
| --- | --- |
| TNF-αF | GGTGCCTATGTCTCAGCCTCTT |
| TNF-αR | GCCATAGAACTGATGAGAGGGAG |
| IL-6F | GAGGATACCACTCCCAACAGACC |
| IL-6R | AAGTGCATCATCGTTGTTCATACA |
| IL-1βF | AATCTCACAGCAGCACATC |
| IL-1βR | AGCAGGTTATCATCATCATCC |
| MyD88F | ACTGATGCGGAGCCAGATTC |
| MyD88R | TGGGAGGAAAGGCAGTCCTA |
| β-actinF | GGCTGTATTCCCCTCCATCG |
| β-actinR | CCAGTTGGTAACAATGCCATGT |
| U6F | GGAACGATACAGAGAAGATTAGC |
| U6R | TGGAACGCTTCACGAATTTGCG |
| miR-340-5p | TTATAAAGCAATGAGACTGATT |
| miR-141-3p | TAACACTGTCTGGTAAAGATGG |
| miR-126b-5p | ATTATTACTCACGGTACGAGTT |
| miR-199b-5p | CCCAGTGTTTAGACTACCTGTTC |
| miR-199a-5p | CCCAGTGTTCAGACTACCTGTTC |
| miR-136-5p | ACTCCATTTGTTTTGATGATGG |
| miR-10b-5p | TACCCTGTAGAACCGAATTTGTG |
| miR-221-5p | ACCTGGCATACAATGTAGATTTCTGT |
| miR-21a-3p | CAACAGCAGTCGATGGGCTGTC |
| miR-125a-5p | TCCCTGAGACCCTTTAACCTGTGA |
| miR-125b-5p | TCCCTGAGACCCTAACTTGTGA |
| miR-149-5p | TCTGGCTCCGTGTCTTCACTCCC |
| miR-18a-3p | ACTGCCCTAAGTGCTCCTTCTG |
| miR-3470a | TCACTTTGTAGACCAGGCTGG |
| miR-466i-5p | TGTGTGTGTGTGTGTGTGTG |
| miR-504-5p | AGACCCTGGTCTGCACTCTATC |
| miR-664-5p | CTGGCTGGGGAAAATGACTGG |
| miR-674-5p | GCACTGAGATGGGAGTGGTGTA |
| miR-8112 | TCTCCGCCACCTCCACCGCA |
