# Supplementary material for: MyD88 inhibitor TJ-M2010-5 alleviates spleen impairment and inflammation by inhibiting the PI3K/miR-136-5p/AKT3 pathway in the early infection of Trichinella spiralis
Source: Vet Res. 2025 Feb 4;56:28. doi: 10.1186/s13567-025-01459-2 (PMC11796171; doi:10.1186/s13567-025-01459-2)
Supplement: Supplementary file 2 — Additional file 2. Antibodies used in this study. [file 13567_2025_1459_MOESM2_ESM.docx]

**Additional file 2 Antibodies used in this study.**

| **Antibody** | **Dilution** | **Company** |
| --- | --- | --- |
| β-actin | 1:1000 | Affinity Biosciences, Changzhou, China |
| MyD88 | 1:1000 | Bioss, Beijing, China |
| tubulin | 1:1000 | Affinity Biosciences, Changzhou, China |
| LaminB | 1:1000 | ZENBIO, Chengdu, China |
| GAPDH | 1:1000 | Servicebio, Wuhan, China |
| p-PI3K | 1:1000 | Cell Signaling Technology, Danvers, MA, USA |
| PI3K | 1:1000 | Proteintech Group, Chicago, IL, USA |
| p-AKT | 1:1000 | Cell Signaling Technology, Danvers, MA, USA |
| AKT | 1:1000 | Cell Signaling Technology, Danvers, MA, USA |
| iNOS | 1:1000 | Affinity Biosciences, Changzhou, China |
| Goat anti rabbit  antibody | 1:5000 | Affinity Biosciences, Changzhou, China |
| Goat anti mouse  antibody | 1:5000 | Affinity Biosciences, Changzhou, China |
