# Supplementary figures and images for: MyD88 inhibitor TJ-M2010-5 alleviates spleen impairment and inflammation by inhibiting the PI3K/miR-136-5p/AKT3 pathway in the early infection of Trichinella spiralis
Source: Vet Res. 2025 Feb 4;56:28. doi: 10.1186/s13567-025-01459-2 (PMC11796171; doi:10.1186/s13567-025-01459-2)

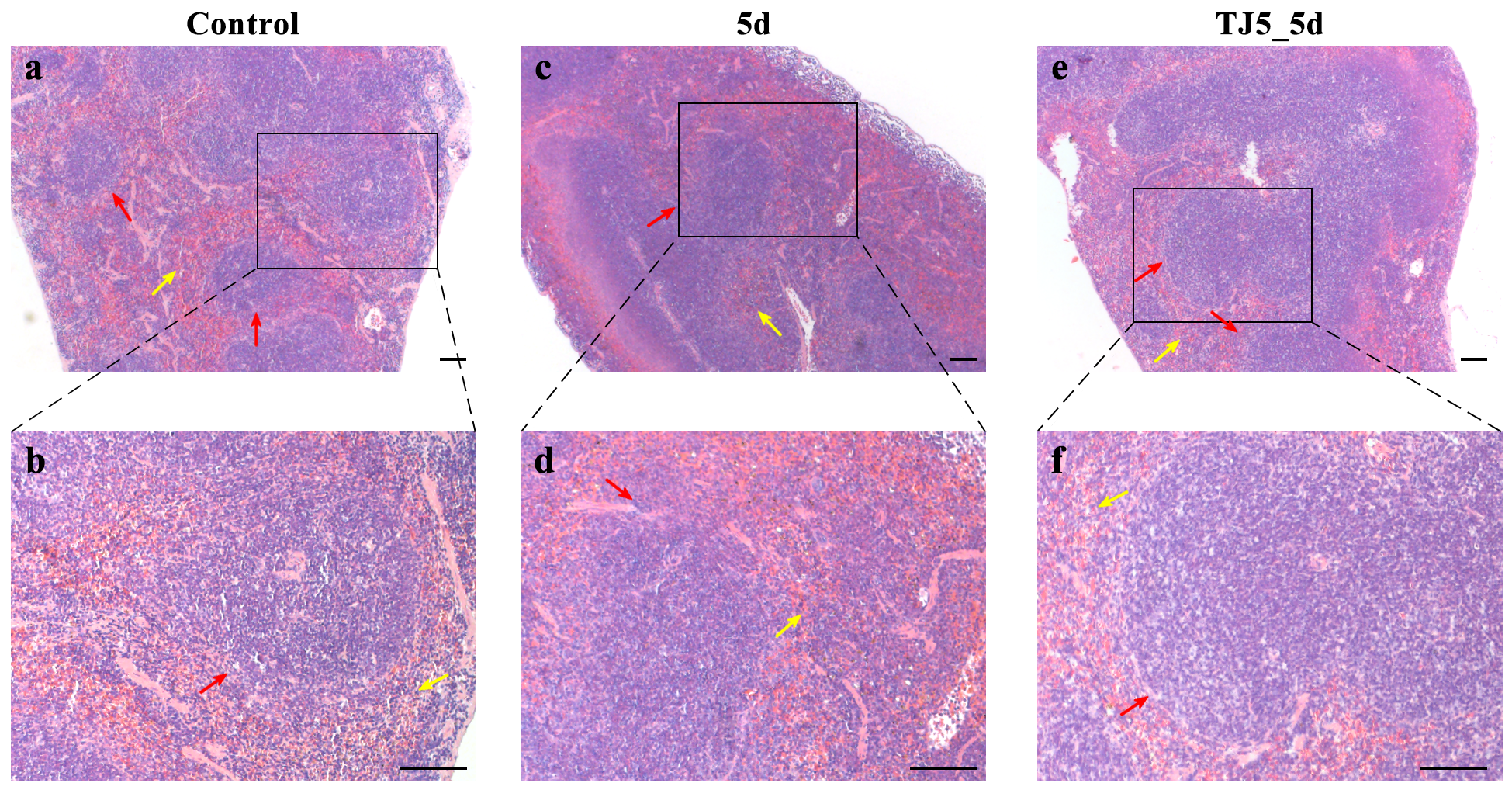

Supplement: Supplementary file 3 — Additional file 3. TJ-M2010-5 protective effect on spleen impairment in T. spiralis infected mice. [file 13567_2025_1459_MOESM3_ESM.tif]

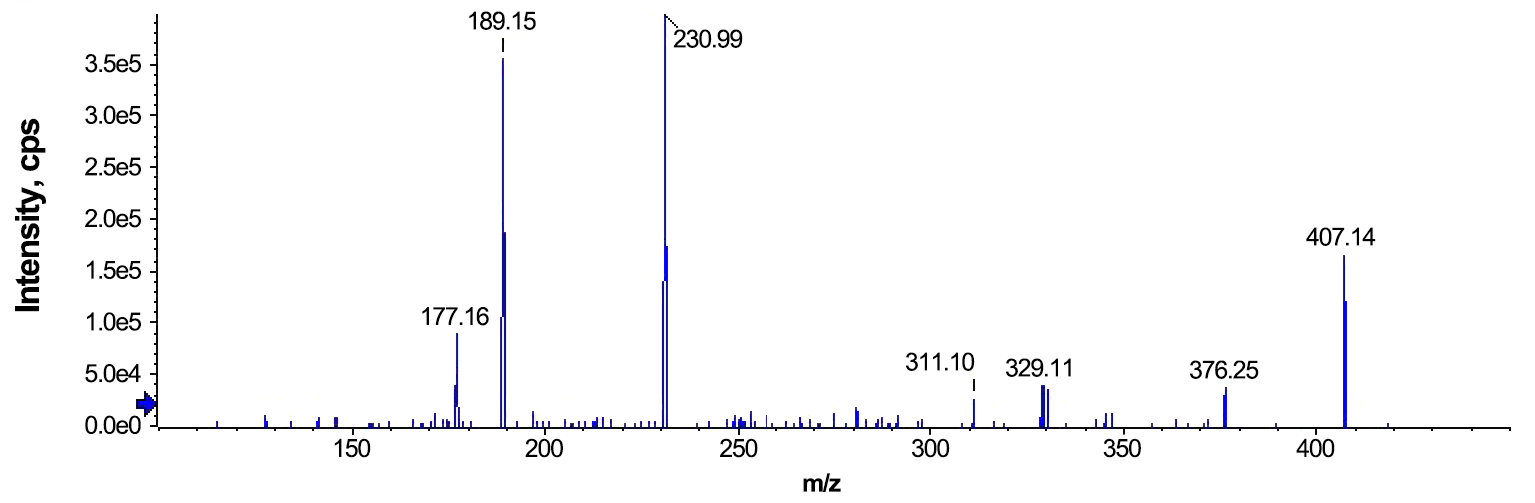

Supplement: Supplementary file 4 — Additional file 4. Positive mode electrospray ionisation (ESI) mass spectrum of TJ-M2010-5. [file 13567_2025_1459_MOESM4_ESM.tif]

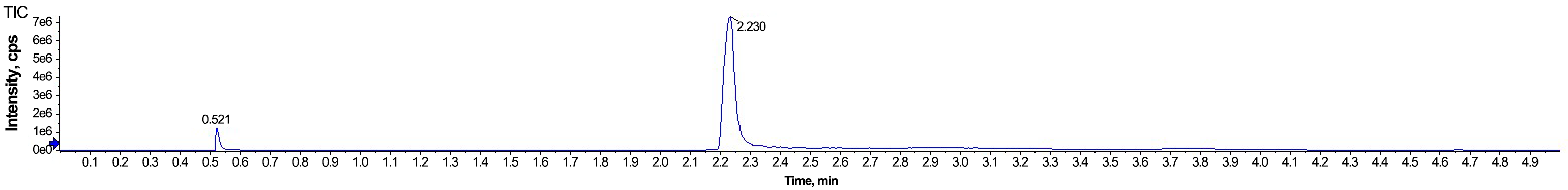

Supplement: Supplementary file 5 — Additional file 5. Total ion chromatogram (TIC) of TJ-M2010-5. [file 13567_2025_1459_MOESM5_ESM.tif]

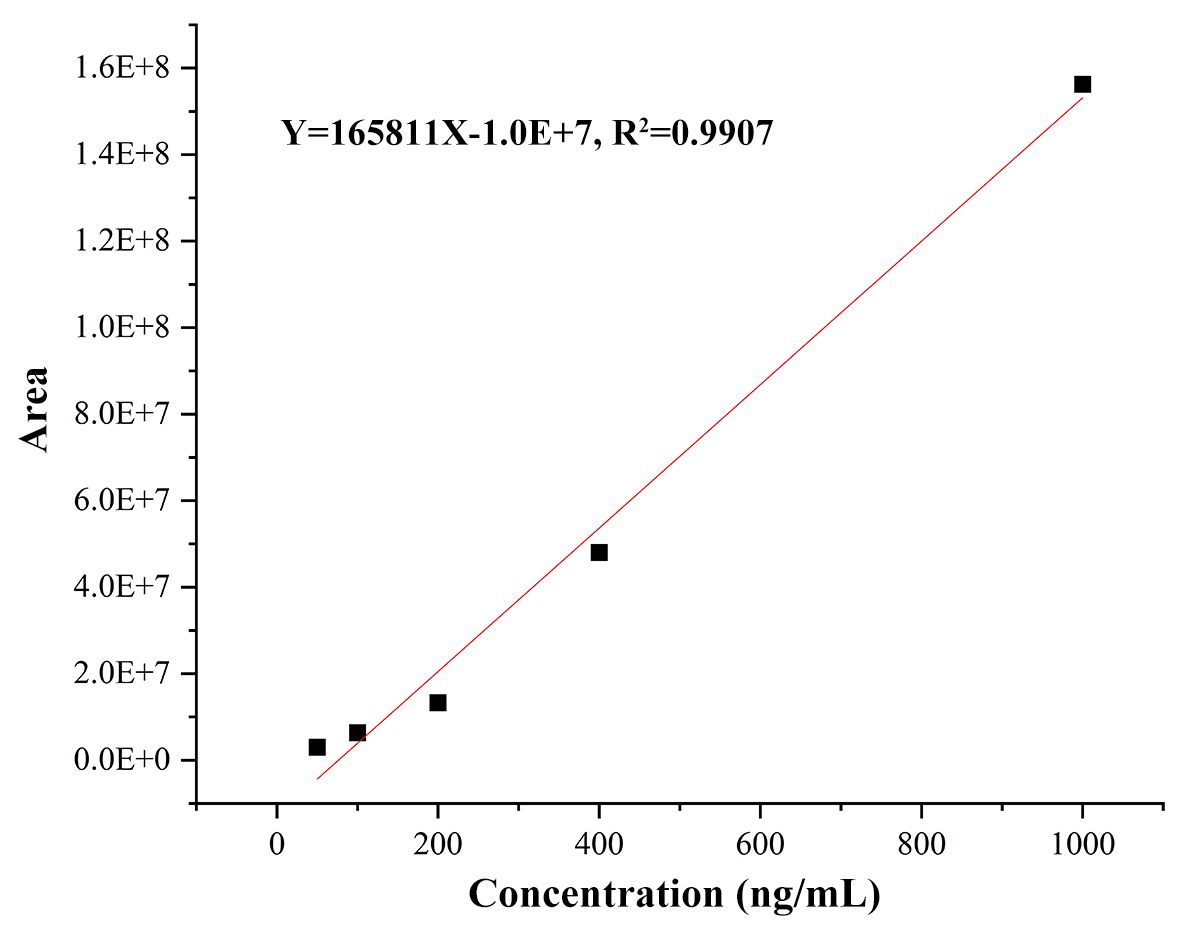

Supplement: Supplementary file 6 — Additional file 6. Calibration curve, correlation coefficients and linear ranges of TJ-M2010-5. [file 13567_2025_1459_MOESM6_ESM.tif]
